# The diversity of substrates for plant respiration and how to optimize their use

**DOI:** 10.1093/plphys/kiac599

**Published:** 2022-12-27

**Authors:** Xuyen H Le, A Harvey Millar

**Affiliations:** School of Molecular Sciences, The University of Western Australia, 35 Stirling Highway, Crawley, Perth 6009, Australia; The ARC Centre of Excellence in Plant Energy Biology, The University of Western Australia, 35 Stirling Highway, Crawley, Perth 6009, Australia; School of Molecular Sciences, The University of Western Australia, 35 Stirling Highway, Crawley, Perth 6009, Australia; The ARC Centre of Excellence in Plant Energy Biology, The University of Western Australia, 35 Stirling Highway, Crawley, Perth 6009, Australia

## Abstract

Plant respiration is a foundational biological process with the potential to be optimized to improve crop yield. To understand and manipulate the outputs of respiration, the inputs of respiration—respiratory substrates—need to be probed in detail. Mitochondria house substrate catabolic pathways and respiratory machinery, so transport into and out of these organelles plays an important role in committing substrates to respiration. The large number of mitochondrial carriers and catabolic pathways that remain unidentified hinder this process and lead to confusion about the identity of direct and indirect respiratory substrates in plants. The sources and usage of respiratory substrates vary and are increasing found to be highly regulated based on cellular processes and environmental factors. This review covers the use of direct respiratory substrates following transport through mitochondrial carriers and catabolism under normal and stressed conditions. We suggest the introduction of enzymes not currently found in plant mitochondria to enable serine and acetate to be direct respiratory substrates in plants. We also compare respiratory substrates by assessing energetic yields, availability in cells, and their full or partial oxidation during cell catabolism. This information can assist in decisions to use synthetic biology approaches to alter the range of respiratory substrates in plants. As a result, respiration could be optimized by introducing, improving, or controlling specific mitochondrial transporters and mitochondrial catabolic pathways.

## Teaser

Plant oxidation of respiratory substrates is regulated by mitochondrial transport and metabolic processes; modifications may enable oxidation of alternative substrates and improve efficiency.

## Introduction

CO_2_ is reduced and assimilated into carbon rich molecules via photosynthesis, 30%–60% of which are oxidized by respiration and released back to the environment as CO_2_ during the plant lifecycle (Raich et al., 2014; Amthor et al., 2019). Beyond this simple comparison, however, the systems that underlie photosynthetic and respiratory capacity deviate markedly. The substrate for photosynthesis is singular, CO_2_, and even though there are different ways that CO_2_ is introduced to chloroplasts in C3, C4, and Crassulacean acid metabolism (CAM) plants (Treves et al., 2022), there is inherently no real flexibility in the input. On the other hand, the inputs to respiration are much more complicated as post-photosynthetic metabolism creates a wide range of reduced carbon molecules that have been reported to act as respiratory fuel sources including sugars, amino acids, organic acids, fatty acids, chlorophylls, and other carbon compounds (Hörtensteiner and Kräutler, 2011; O'Leary et al., 2019). Respiration begins with a substrate selection inside cells through metabolic enzyme abundances and regulation which might not be optimized by evolution for the environments and conditions commonly encountered by many plants in managed environments today. Direct substrates for respiration are defined here as those transported into the mitochondrial matrix and metabolized to generate reducing equivalents (NADH and FADH_2_). These can be used to drive the electron transport chain (ETC) and oxidative phosphorylation in mitochondria with or without CO_2_ release. There are also other compounds external to mitochondria that can be metabolized to provide tricarboxylic acid (TCA) cycle intermediates or NAD(P)H to mitochondria; however, they will not be the focus of this review.

To further complicate the landscape of respiratory substrates, partial oxidation of respiratory substrates and the operation of non-phosphorylating bypasses in the ETC limit the efficiency of respiration in generating ATP (Millar et al., 2011). Externally facing NAD(P)H dehydrogenases on the mitochondrial inner membrane effectively allow excess reductant from any cytosolic process to be used as a respiratory substrate, but these pathways are reviewed elsewhere (Rasmusson et al., 2008). Additionally, there remains a considerable debate about what the ideal balance is between “maintenance” and “growth” respiration, and even what these terms mean. Whether or not there is futile or wasteful respiration in plants and which substrates drive its rate is a matter of debate (Padmasree et al., 2002; Araújo et al., 2014; Vanlerberghe et al., 2020; Scafaro et al., 2021). The notion of “optimal respiration” that can balance cellular redox states, contribute to ideal photosynthetic capacity, and in turn support growth, biomass, and yield can be theorized but is hard to quantify in practice (Tcherkez et al., 2017; O'Leary et al., 2019). Ultimately, the problem is that respiration rate is not one parameter but sum of the oxidation of various cellular resources and the various benefits extracted during the process, both centered around the operation of mitochondrial metabolism and oxidative phosphorylation.

Our knowledge of which respiratory substrates are the major contributors throughout the diurnal cycle, under stress conditions, and across plant development is improving but remains incomplete. Our insight into how plant cells prioritize carbon sources to break down for energy, or to use for biosynthesis and storage, and when to switch, is still elementary. This Update Review aims to provide an update on recent discoveries in the use of respiratory substrates under normal and stressed conditions and delineate the gaps in our current understanding as we seek respiratory optimization in plants.

## The flexible use of major substrates for plant respiration

### Pyruvate as a hub for respiratory substrate choices

**Figure 1:**
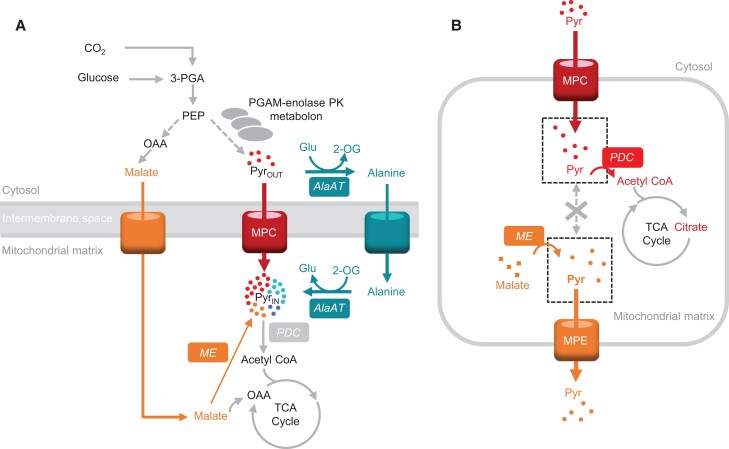
The flexibility and regulation of pyruvate use as a respiratory substrate. A, Multiplicity of pyruvate-supplying pathways to support mitochondrial respiration in Arabidopsis seedlings. B, The imported pyruvate pool does not freely mix with the ME-derived pyruvate pool and is prioritized for oxidation by PDC in isolated mitochondria. AlaAT, alanine aminotransferase; ME, malic enzyme; MPC, mitochondrial pyruvate carrier; MPE, mitochondrial pyruvate exporter; PDC, pyruvate dehydrogenase complex; PGAM-enolase-PK, phosphoglycerate mutase-enolase-pyruvate kinase metabolon. Metabolites: Pyr, pyruvate; Glu, glutamate; 2-OG, 2-oxoglutarate; OAA, oxaloacetate; 3-PGA, 3-phosphoglycerate.

Pyruvate is widely considered the primary substrate that drives eukaryotic mitochondrial respiration rate. Pyruvate is transported to the mitochondrial matrix via mitochondrial pyruvate carriers (MPCs) residing in the inner mitochondrial membrane and the first step of pyruvate oxidation is carried out in the matrix by the mitochondrial pyruvate dehydrogenase complex (mtPDC) yielding acetyl-CoA, NADH, and CO_2_. Knocking out either MPCs (Gray et al., 2014; McCommis et al., 2015) or the mitochondria pyruvate dehydrogenase complex (Johnson et al., 2001; Gopal et al., 2018) has a detrimental effect on mammalian cell development. In comparison, the plasticity of pyruvate provision to respiration in plant mitochondria appears to be greater. While mutations in PDC subunits in Arabidopsis (*Arabidopsis thaliana*) cause retardation in organ size and root length (LeClere et al., 2004; Taylor et al., 2004; Quint et al., 2009; Yu et al., 2012; Song and Liu, 2015), abolition of pyruvate mitochondrial transport does not grossly affect plant vegetative growth phenotypes (Li et al., 2014; Wang et al., 2014; He et al., 2019). Detailed biochemical study of mutants in transporters and metabolic enzymes in Arabidopsis show that while pyruvate imported via MPC is a major pathway providing pyruvate to mitochondria (Le et al., 2021), at least two other pathways work co-operatively with MPC to provide sufficient pyruvate for plant respiration and growth (Le et al., 2021; Figure 1A). These include pyruvate production from alanine and malate inside the mitochondria, internal pyruvate production via NAD-dependent malic enzyme (NAD-ME), and an alanine shuttle via reversible alanine aminotransferase (AlaAT) reactions (Le et al., 2021) that are situated in the cytosol and the mitochondrial matrix (Liepman and Olsen, 2003). This means that alanine and malate act as direct respiratory substrates via their conversions to pyruvate inside plant mitochondria. The loss of any one of these systems does not affect vegetative phenotypes in Arabidopsis (Le et al., 2021).

#### Pyruvate production provision via a glycolytic metabolon

Respiration at night relies largely on catabolism of daily replenished starch reserves in leaves and on sugars transported from photosynthesizing tissues to produce respiratory pyruvate in non-photosynthesizing plant tissues (MacNeill et al., 2017). Cytosolic glycolysis is the primary feeder of pyruvate to mitochondrial respiration via MPC (Flores-Tornero et al., 2017). The primary source of this cytosolic pyruvate in photosynthesizing tissues in the light is 3-PGA, produced via the Calvin–Benson cycle and exported from chloroplasts to the cytosol via the triose phosphate translocator (TPT; Walters et al., 2004; Schmitz et al., 2012). Even though 3-PGA does not enter the mitochondria, its catabolism is tightly linked to the availability of pyruvate for import. There is evidence that TPT physically associates with a phosphoglycerate mutase (PGAM)-enolase-pyruvate kinase metabolon (Zhang et al., 2020), which is in contact with the mitochondrial outer membrane (Graham et al., 2007). A physical association of chloroplasts and mitochondria, by this or other means, also promotes the direct usage of plastid 3-PGA to provide pyruvate, via MPC, for mitochondrial respiration in the light (Zhang et al., 2020).

#### Pyruvate from alanine via AlaAT

Alanine is converted into pyruvate via a transamination reaction catalyzed by AlaAT coupled with the amination of 2-oxoglutarate (2-OG) into glutamate (Miyashita et al., 2007). The catabolism of alanine does not generate extra reducing equivalents; however, it ensures the supply of pyruvate from versatile and available carbon and nitrogen storage molecules. In the absence of imported pyruvate and pyruvate made from malate, AlaAT alone can support relatively normal growth in Arabidopsis, suggesting it can carry considerable respiratory flux under normal conditions (Le et al., 2021).

Coupled with the fact that alanine biosynthesis is energetically inexpensive compared with biosynthesis of other amino acids (Arnold et al., 2015), the versatility of alanine in metabolic pathways could make it the perfect balance of respiratory substrate and nitrogen store. *In vivo*^13^C labeling patterns show that pyruvate is readily converted to alanine via alanine aminotransferase (AlaAT; Tcherkez et al., 2005) in the light (Tcherkez et al., 2017) when plants are fed with labeled glucose or pyruvate. Alanine concentration correlates better than any other metabolite with respiratory rate in different Arabidopsis ecotypes, and exogenous alanine addition can stimulate respiration in the dark more readily than pyruvate, indicating a disequilibrium or distinct subcellular localization of pyruvate and alanine pools in leaf cells (O'Leary et al., 2017). Plants have evolved metabolic and gene regulatory responses via the Target of Rapamycin (TOR) signaling pathway that act to rapidly metabolize the accumulated alanine (O'Leary et al., 2020). Moreover, alanine to pyruvate interconversion has been shown to be essential in plant hypoxia responses (Good and Muench, 1993; Limami et al., 2008; Rocha et al., 2010; Alexova et al., 2015) and considered the most commonly employed metabolite transported in C4 plants as alanine conserves carbon, nitrogen, and reductants (Mallmann et al., 2014; Rao and Dixon, 2016; Schlüter et al., 2019). Interestingly, over-expression of AlaAT in plants increased biomass and yield in Arabidopsis (McAllister and Good, 2015), rapeseed (*Brassica napus*; Good et al., 2007), and rice (*Oryza sativa*; Shrawat et al., 2008), which suggests that the flexibility of using pyruvate and alanine as respiratory substrates can influence plant yield.

#### Pyruvate from malate via NAD-ME

The oxidation of malate to form pyruvate via NAD-ME results in production of NAD(P)H and CO_2_ and a high respiratory quotient of complete oxidation of malate. This might be one reason why malate is not used as a respiratory substrate via NAD-ME even when MPC and AlaAT are absent, which leads to deficiencies in both shoot and root growth (Le et al., 2021). We have found that pyruvate imported via MPC is prioritized over NAD-ME-derived pyruvate to provide the carbon backbone for citrate production in the TCA cycle and respiration (Le et al., 2022; Figure 1B) due to the substrate channeling-like behavior (Wheeldon et al., 2016) between MPC and PDC (Le et al., 2022). Pyruvate made from imported malate appeared to be prioritized to be exported to support biosynthesis of amino acids or fatty acids in other cell compartments (Le et al., 2021).

The role of NAD-ME as a mitochondrial pyruvate provider for respiration during the diurnal cycle or in specific tissues remains to be confirmed. NAD-ME transcript levels and pathway flux is considerable during seed maturation in soybean (Gerrard Wheeler et al., 2016) and sunflower seeds (Alonso et al., 2007) which might be related to respiration, or increased fatty acid generation from malate in these tissues. Fine mapping of genes responsible for regulating temporal metabolic shifts between plant primary and secondary metabolism has identified NAD-ME1 as a major locus, suggesting a role in biosynthesis ahead of respiration in Arabidopsis (Francisco et al., 2021). In addition, NAD-ME expression is reported to be upregulated during light-dark transitions in C3 plants (Igamberdiev et al., 2001; Lee et al., 2010; Lehmann et al., 2016), suggesting roles for malate oxidation that precede the steady state of starch degradation fueling respiration through glycolysis. In C4 plants such as maize (*Zea mays*), while total NAD-ME activity was decreased by light, it was found that NAD-ME1 expression was decreased while NAD-ME2 expression was increased by light. This could indicate divergent diurnal roles between NAD-ME isoforms (Eprintsev et al., 2020). However, this conclusion requires further verification to deduce the light-dependent role for NAD-ME.

### Stored organic acids (re)-enter the TCA cycle via specific mitochondrial carriers

#### Malate via NAD-malate dehydrogenase

**Figure 2:**
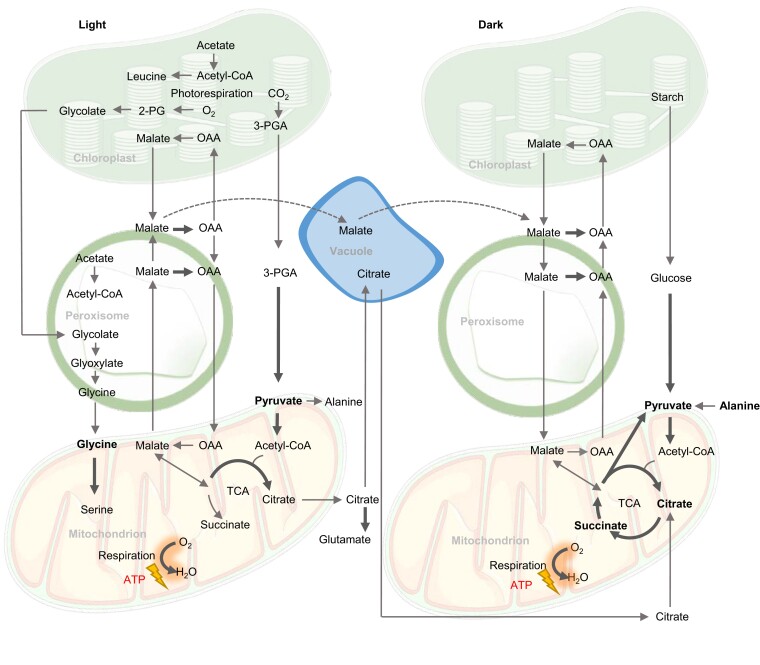
Biochemical pathways of respiratory substrates used in the light and in the dark in plants. The operation of the TCA cycle in a partial mode in the light versus complete mode in the dark is indicated. Bold arrows indicate electron transfer directly or indirectly to the respiratory chain of mitochondria. Bold metabolites are considered direct respiratory substrates.

Malate is one of the main respiratory substrates used at the end of the night when there is a metabolic shift from respiration of starch and sugar toward organic acids (Zell et al., 2010; Figure 2). NAD-malate dehydrogenase (NAD-MDH) is the final enzyme of the TCA cycle and ensures optimal ATP production in the dark to drive cell maintenance (Tomaz et al., 2010). Malate accumulates in the vacuole during the day up to a concentration of 200 mM (Borland et al., 2009) and the loss of this malate pool at the end of the night promotes the usage of amino acids, fatty acids, and even chlorophylls for respiration (Fahnenstich et al., 2007; Maurino and Engqvist, 2015). As well as being a respiratory substrate, malate participates with oxaloacetate (OAA) in a shuttle to influence the NADH/NAD^+^ ratio (Selinski and Scheibe, 2019). This allows it to both indirectly activate multiple components of the respiratory apparatus that are sensitive to high NADH, such as PDC, isocitrate dehydrogenase, and glycine decarboxylase (GDC; Bykova et al., 2014; Igamberdiev et al., 2014; Lindén et al., 2016), and to have various roles in stomata closure, pH, and redox balance (Selinski and Scheibe, 2019). Several historical studies postulated the presence of separate mitochondrial pools of malate for different purposes in castor bean seed and pea leaf mitochondria (Brailsford et al., 1986; Wiskich et al., 1990). In the absence of further evidence, alternative explanations based on the operation of enzymes with low turnover rates but present at high concentrations allow a model of MDH as a buffering enzyme (Bykova et al., 2014; Igamberdiev et al., 2014). This would mean MDH could facilitate the removal of NADH from the proximity of GDC without the need for separate metabolic domains. Our recent study shows there are at least two major pathways to import malate into the mitochondrial matrix, one via dicarboxylate carrier 2 (DIC2) and dependent on citrate-coupled export, and the other via malate carriers with unknown dependencies. This suggests that different transport mechanisms might also be able to support segregated roles of malate inside plant mitochondria (Lee et al., 2021).

#### Citrate

Citrate is a versatile organic acid made by the TCA cycle during both the day and night (Figure 2). It can enter or be made in mitochondria and undergo complete oxidation to generate redox equivalents for ATP production or it can be exported from the mitochondrial matrix. In the light, a non-cyclic form of the TCA cycle generates citrate and exports it for vacuolar storage or amino acid biosynthesis (Eprintsev et al., 2015b). At night, respiration by the cyclic TCA cycle extracts three NADH and one FADH_2_ from the oxidation of every citrate molecule, while a small proportion of the citrate is exported and stored to provide carbon skeletons for glutamate and glutamine synthesis the following day (Cheung et al., 2014; Tcherkez et al., 2017).

Like malate, citrate is stored in the vacuole at high concentration, suggesting its potential to act as a respiratory substrate. Citrate can re-enter mitochondria via citrate importers and pass into the TCA cycle when NADH concentration and glycolytic flux are low (Igamberdiev and Eprintsev, 2016). A homolog of the succinate-fumarate carrier (SFC) displays citrate/isocitrate exchange transport activity as well as succinate-fumarate exchange activity (Brito et al., 2020). The use of mitochondrially generated citrate as a respiratory substrate is mediated by an MDH-citrate synthase-aconitase (MDH-CS-ACO) metabolon which reserves a certain pool of mitochondrial citrate for further oxidation to generate NADH (Huang et al., 2018). There is also evidence in yeast for a separate citrate synthase-citrate exporter metabolon to generate a protected pool of citrate for export from mitochondria (Grigorenko et al., 1990). While the former is yet to be confirmed in plants, the role of DIC2 in importing malate in exchange for mitochondrial citrate could suggest a pool of mitochondrial citrate is exported to generate nicotinamide adenine dinucleotide phosphate (NADPH) in the cytosol to create reducing power in the light for maintenance respiration and secondary metabolism (Lee et al., 2021).

#### Succinate

Succinate can be made from succinyl-CoA which forms a part of the TCA cycle and is coupled with the substrate-level synthesis of ATP inside plant mitochondria (Studart-Guimarães et al., 2007) or can likely be imported into plant mitochondria from the cytosol via one of the DICs (Picault et al., 2002; Palmieri et al., 2008) or SFC (Catoni et al., 2003). The substrates for these transporters have been identified using liposome reconstitution and yeast complementation but have not yet been functionally characterized in plants. Succinate oxidation to fumarate in the mitochondria contributes to respiration directly as it transfers electrons inside complex II to ubiquinone. Succinate also contributes indirectly to the production of malate in the mitochondrial matrix which can be oxidized to OAA and generate NADH (Huang and Millar, 2013; Belt et al., 2017; Restovic et al., 2017; Figure 2).

**Figure 3:**
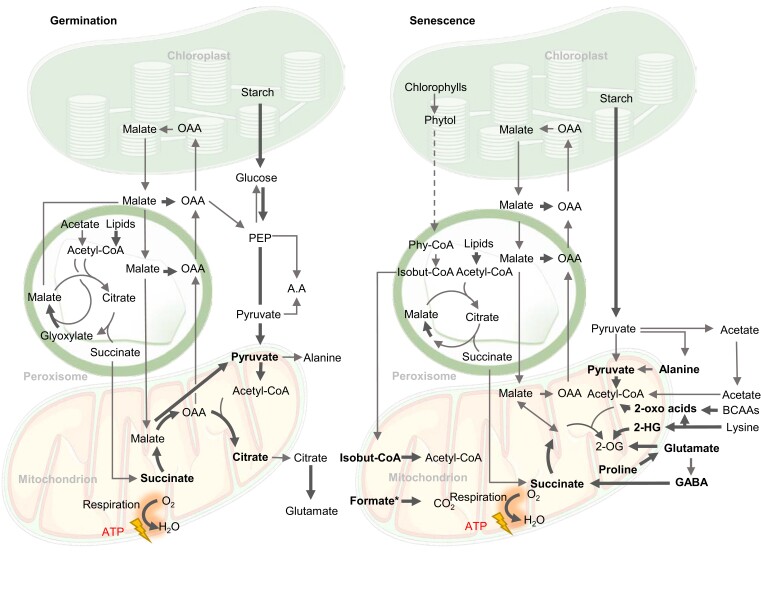
Biochemical pathways of respiratory substrates used during germination and senescence in plants. Bold arrows indicate reactions leading directly or indirectly to electron transfer to the respiratory chain of mitochondria. Bold metabolites are considered direct respiratory substrates. 2-OG, 2-oxoglutarate; 2-HG, 2-hydroxyglutarate; 3PGA, 3-phosphoglyceric acid; A.A, amino acids; BCAAs, branched-chain amino acids; isobut-CoA, isobutyryl-CoA; PEP, phosphoenolpyruvate; OAA, oxaloacetate; Phy-CoA, phytanoyl-CoA. In oily seed germination, malate from glyoxylate cycle and TCA cycle generates glucose, while in starchy seeds glucose comes from starch. The dotted arrow from phytol to phytanoyl-CoA indicates the uncertainty of cellular location of the steps involved. Formate* accumulates under plant stress from an unknown combination of serine degradation during photorespiration, methanol metabolism, or from glyoxylate (Alekseeva et al., 2011).

Succinate can also be made by the glyoxylate cycle in the peroxisome from acetyl-CoA via isocitrate lyase (ICL). The glyoxylate cycle is important for lipid mobilization during germination of oil-storing seeds but also operates at a lower flux in leaves in the light (Eprintsev et al., 2015a). The glyoxylate cycle takes acetyl-CoA from lipid oxidation to produce succinate and subsequently malate via malate synthase (MLS). Malate/OAA is exported to the cytosol for gluconeogenesis via phosphoenolpyruvate carboxykinase (Figure 3; Cornah et al., 2004; Eastmond et al., 2015). SFC is highly expressed in 2-day-old Arabidopsis seedings but declines in expression in cotyledons. This suggests a role of mitochondrial succinate transport in lipid mobilization (Catoni et al., 2003). Succinate made in the glyoxylate cycle can enter TCA cycle to produce malate/OAA for gluconeogenesis and concurrently produce NADH for oxidative phosphorylation and ATP production (Eastmond and Graham, 2001; Thorneycroft et al., 2001). Taken together, the glyoxylate cycle bypasses the decarboxylation steps of the TCA cycle so that carbon from seed oil can be converted by its non-decarboxylating steps to starch via gluconeogenesis or into amino acids via transamination during germination (Graham, 2008). Apart from its role in germination, ICL gene expression was induced in senescent barley (*Hordeum vulgare*) leaves (Gut and Matile, 1988; Eastmond and Graham, 2001) and in Arabidopsis under salt stress (Yuenyong et al., 2019), suggesting its role in providing TCA cycle intermediates to cope with environmental stress. The activity of 2-OG dehydrogenase complex (OGDH) is disrupted under saline conditions (Che-Othman et al., 2020), hence the increased metabolic flux through ICL can bypass OGDH and provide succinate to re-enter mitochondria for respiration.

### Low-cost amino acids are respiratory substrates under normal conditions

#### Glycine

In the light, oxygenation of RuBP by Rubisco produces one molecule of 3-PGA and one molecule of 2-phosphoglycolate (2-PG; Flügel et al., 2017). The 2-PG is converted to glycolate, which is shuttled out of chloroplasts to the peroxisomes where it is converted to glyoxylate and then aminated to glycine in the first steps of the photorespiratory cycle (Charton et al., 2019). Photorespiration can account for ∼20% of assimilated CO_2_ under current CO_2_ concentrations (Erb and Zarzycki, 2018) and even more at low CO_2_ concentrations (Sharkey, 1988), hence glycine flux to mitochondria in the light is substantial. Glycine transport into plant mitochondria is inhibited by mersalyl and glycine analogs (Walker et al., 1982); however, the carrier involved is still unknown, despite the discovery and characterization of glycine carriers in human and yeast mitochondria (Lunetti et al., 2016). Imported glycine is converted to serine in plant mitochondria via GDC and serine hydroxymethyltransferase, releasing a molecule each of CO_2_, NH_4_^+^, and NADH. The metabolism of mitochondrial photorespiratory products inhibits respiration of other substrates in the light via its effect on the matrix NADH/NAD^+^ ratio (Bykova et al., 2005).

A large proportion of this serine is recycled back to the peroxisome to regenerate 3-PGA for the cost of one ATP, but the amount of NADH available for oxidative phosphorylation and ATP production from glycine oxidation is still likely to be positive. The exact amount of NADH used for ATP production in the mitochondria will depend on the proportion transferred to the cytosol by malate exchange and the engagement of non-coupled respiratory pathways during photorespiration. GDC knockdown mutants respire faster in the dark due to greater accumulation of TCA cycle intermediates in the light. Alternative decarboxylation of photorespiratory intermediates may complement the loss of mitochondrial NADH generation in these mutants (Lin et al., 2016). Under normal conditions in C3 plants, glycine oxidation is the main pathway for mitochondrial redox equivalent generation in the light; >50% of mitochondrial NADH and even more under high light conditions (Eisenhut et al., 2019; Giuliani et al., 2019; Figure 2). Similar to alanine, glycine is considered an energetically low-cost amino acid for cells to make compared with other amino acids (Arnold et al., 2015) and is readily available in the light. Glycine is the pre-eminent example of optimized use of photosynthetic by-products to generate NADH in the light while alanine might be considered the most versatile substrate and a flexible and direct source of mitochondrial pyruvate in the dark.

### Higher-cost amino acids and formate as respiratory substrates under stress

Most amino acids made in leaves or recycled from existing proteins are not oxidized by respiration at a high rate (Hildebrandt et al., 2015; Hildebrandt, 2018) as amino acid synthesis, transport, and incorporation into proteins is energetically expensive (Arnold et al., 2015). However, under conditions of insufficient carbohydrate supply, plants rely on amino acids from protein degradation as alternative respiratory substrates (Araújo et al., 2011b; Millar et al., 2011). The amino acids most used as alternative respiratory substrates belong to a moderately costly group (proline, glutamate, branched-chain amino acids [BCAAs]) (Arnold et al., 2015; Figure 3), while high-cost amino acids such as arginine, histidine, tyrosine, tryptophan, and phenylalanine generally serve as precursors for secondary metabolites (Batista-Silva et al., 2019). Their catabolism does not occur as fast as proline, BCAAs, or lysine during stress recovery (Batista-Silva et al., 2019) suggesting it might only occur when other respiratory substrates are depleted. Surprisingly, the catabolic machinery responsible for degradation of most aromatic amino acids has yet to be determined in detail in plants (Hildebrandt et al., 2015). In addition, formate can be generated during abiotic stress and is oxidized in mitochondria (Alekseeva et al., 2011).

#### Proline

**Figure 4:**
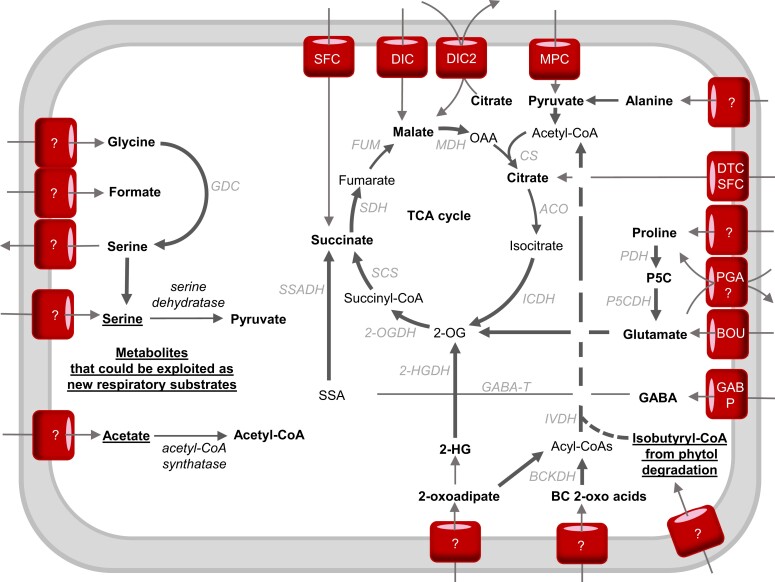
The interplay between mitochondrial transport and catabolic pathways in the use of respiratory substrates in plants. Bold arrows indicate electron-transfer reactions. Bold metabolites are considered respiratory substrates. Underlined metabolites have the potential to become respiratory substrates if proposed mitochondrial carriers and catabolizing enzymes exist or are artificially introduced into mitochondria. The dotted line from acyl-CoAs to acetyl-CoA indicates that some steps might not occur in the mitochondria (Hildebrandt et al., 2015). Enzymes: 2-OGDH, 2-oxoglutarate dehydrogenase; 2-HGDH, 2-hydroxyglutarate dehydrogenase; ACO, aconitase; AlaAT, alanine aminotransferase; BOU, A bout de soufflé carrier; CS, citrate synthase; BCKDH, branched-chain amino acid dehydrogenase; DIC, dicarboxylate carrier; FUM, fumarase; GABP, GABA permease; GABA-T, GABA transaminase; GDC, glycine decarboxylase; ICDH, isocitrate dehydrogenase; IVDH, isovaleryl-CoA dehydrogenase; MPC, mitochondrial pyruvate carrier; DTC, di/tricarboxylate carrier; MDH, malate dehydrogenase; ME, malic enzyme; MPC, mitochondrial pyruvate carrier; PGA, proline-glutamate antiporter; PDH, proline dehydrogenase; P5CDH, P5C dehydrogenase; PDC, pyruvate dehydrogenase complex; SCS, succinyl-CoA synthetase; SDH, succinate dehydrogenase; SFC, succinate-fumarate carrier; SSADH, succinic semialdehyde dehydrogenase. Metabolites: 2-OG, 2-oxoglutarate; OAA, oxaloacetate; 2-HG, 2-hydroxyglutarate; P5C, pyrroline-5-carboxylate; SSA, succinic semialdehyde.

Proline is well known to accumulate in high amounts during salt and osmotic stress, sometimes >100-fold compared with during control conditions (Verbruggen and Hermans, 2008; Jacoby et al., 2011). Proline plays important roles in osmoprotection (Hossain et al., 2019) and reactive oxygen species (ROS) defense (Signorelli et al., 2014, 2015). After periods of osmotic stress, proline is removed by catabolism inside mitochondria. Plant mitochondria contain proline dehydrogenase (PDH) and 1-pyrroline-5-carboxylate dehydrogenase to catalyze this catabolism but the identity of proline transporters in mitochondria is still obscure. Based on kinetic studies in isolated mitochondria there are likely to be two proline carriers, one solely for proline and one for a proline-glutamate antiporter (Di Martino et al., 2006; Figure 4). Proline acts as a respiratory substrate in intact plant tissues and isolated plant mitochondria with PDH providing electrons to the ubiquinone pool directly (Schertl and Braun, 2014; O'Leary et al., 2020). A large respiratory flux during proline catabolism can saturate the ETC, exceeding the capacity and energy demand of plant cells and inducing the expression of non-phosphorylating bypasses such as alternative oxidase (AOX) (Oh et al., 2021). The loss of expression of AOX does not abolish proline respiration but instead, it causes increased ROS generation and a prolonged recovery period for plants following proline accumulation due to salt stress (Oh et al., 2021). This suggests that AOX allows faster and safer oxidation of proline to ensure optimal photosynthetic capacity without damaging cell components (Oh et al., 2021). On the other hand, during dark-induced senescence, proline oxidation has been shown to be important to generate glutamate and the electron flux passes through the cytochrome pathway for ATP synthesis (Cabassa-Hourton et al., 2016; Launay et al., 2019).

#### Glutamate and GABA shunt

Glutamate is a major amino acid connecting carbon and nitrogen metabolism (Grzechowiak et al., 2020) especially following germination (Day et al., 1988) and plays an important signaling role for nitrogen and carbon status in plant cells (Qiu et al., 2020). Glutamate is probably imported into plant mitochondria via A BOUT DE SOUFFLE (BOU) which is homologous to the yeast mitochondrial glutamate transporter protein Ycm2p (Eisenhut et al., 2013; Porcelli et al., 2018; Figure 4). Glutamate metabolism inside plant mitochondria is well understood. Glutamate can act as a substrate in isolated mitochondria through its oxidation by glutamate dehydrogenase (GDH) leading to 2-OG, NADH, NH_4_^+^, and CO_2_. In theory, GDH is reversible, but ^15^N-nuclear magnetic resonance experiments *in planta* confirmed that GDH mainly operates in the direction of deaminating glutamate to 2-OG to fuel the TCA cycle, especially when carbon becomes the limiting factor (Labboun et al., 2009), and it generates NAD(P)H in mitochondria (Fontaine et al., 2012).

Alternatively, cytosolic glutamate can be used as a source of γ-aminobutyric acid (GABA) under stress conditions via glutamate decarboxylase in the cytosol (Qiu et al., 2020). GABA is then transported in mitochondria via GABA permease (GABP) where GABA shunt enzymes make succinate to enter the TCA cycle and produce NADH (Michaeli et al., 2011; Figure 4). The GABA shunt is activated in a wide range of plant stresses including starvation, salinity, and hypoxia (Rolletschek et al., 2011; António et al., 2016; Wang et al., 2017; Che-Othman et al., 2020; Jethva et al., 2022). Under conditions of limited carbon supply, the *gabp* mutant displayed growth abnormalities and ∼20% decrease in CO_2_ evolution compared with that in wild-type plants following incubation in labeled GABA (Michaeli et al., 2011). This proves the role of the mitochondrial GABA pool as an alternative respiratory substrate (Bandehagh and Taylor, 2020; Figure 3). The activity of PDC and OGDH has been shown to be inhibited by salinity stress, inducing the GABA shunt (Che-Othman et al., 2020). The GABA shunt helps to overcome this inhibition by bypassing these salt-sensitive enzymes (Che-Othman et al., 2020). It provides an alternative carbon source to facilitate the increased respiration of wheat leaves in order to provide energy for metabolic processes such as ion exclusion, synthesis of compatible solutes, and detoxification of ROS (Dissanayake et al., 2022).

### Branched-chain amino acids

**Figure 5:**
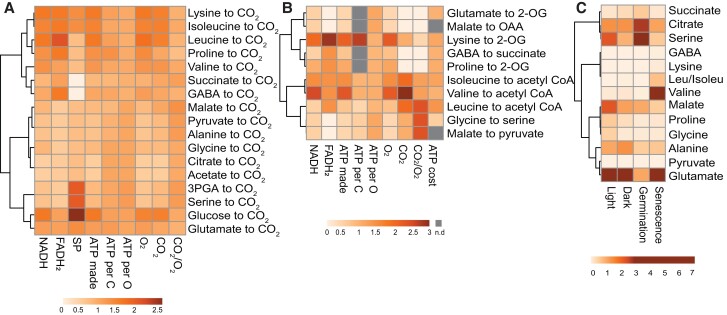
Clustering of respiratory substrates based on their energetic characteristics, stoichiometry of catabolic products, and abundance. A, Complete oxidation of a range of the respiratory substrates mentioned. B, Partial oxidation of mainly amino acids to TCA cycle metabolites. The ATP cost data were retrieved from (Arnold et al., 2015). C, Leaf metabolite content in different conditions. The metabolite amount data in the light (mid-day), dark (midnight), and during senescence (10-day dark-induced) was retrieved from (Lee et al., 2021) and germination metabolite data is retrieved from (Fait et al., 2006). All other parameters were calculated from the catabolism of each substrate. Data were column centered before generating heatmaps using Clusvis Heatmap, values are fold difference to column average for visualization. See Supplemental Table 1 for actual values. n.d., not determined; OAA, oxaloacetate; GABA, gamma-aminobutyric acid; 2-OG, 2-oxoglutarate; 3-PGA, 3-phosphoglyceric acid; SP, substrate level phosphorylation.

The degradation pathways of the BCAAs valine, leucine, and isoleucine have been identified as essential factors for plant survival of sugar starvation (Hirota et al., 2018). The first step of BCAA catabolism mainly occurs in the cytosol. Only one out of six BCAA transaminases have been located in mitochondria, most likely the leucine transaminase (Binder, 2010). Leucine is an effective alternative substrate for respiration owing to its low respiratory quotient compared with other BCAAs and many enzymes required for leucine degradation are present in plant mitochondria (Figure 5). Key enzymes of BCAA metabolism such as branched chain α-keto acid dehydrogenase complex and isovaleryl-CoA dehydrogenase (IVDH) are located inside mitochondria. These enzymes either generate NADH or transfer electrons from α-keto-acids to the ubiquinone pool via the electron-transfer flavoprotein/electron-transfer flavoprotein:ubiquinone oxidoreductase complex (ETF/ETFQO; Kochevenko et al., 2012). Based on our definition of direct substrates, branched chain α-keto-acids are considered respiratory substrates rather than BCAAs themselves. These α-keto-acids require a set of transporters to translocate them to the mitochondrial matrix, which await identification. The regulation of BCAA catabolism is well studied and involves a sucrose non-fermenting 1-related protein kinase 1 (SNRK) bZIP signal transduction pathway which directly regulates the promoter of ETFQO under carbon starvation (Cavalcanti et al., 2017; Pedrotti et al., 2018). Knocking out ETFQO-1, SNRK, BZIP, and other BCAA catabolism enzymes such as IVDH decreased respiration rate, increased rates of senescence, decreased leaf quantum yield, and caused plants to be more sensitive to extended darkness (Pedrotti et al., 2018). BCAA catabolism can also generate acetyl-CoA in the cytosol or mitochondria which also fuels the TCA cycle (Hildebrandt et al., 2015; Arruda and Barreto, 2020). BCAA accumulation is also important during salt stress (Huang and Jander, 2017; Batista-Silva et al., 2019) and BCAA catabolism mutants are more drought-sensitive compared with wild-type plants (Engqvist et al., 2011; Pires et al., 2016). BCAAs are toxic to plant growth, hence the cellular priority to quickly metabolize them after stress (Pires et al., 2016; Batista-Silva et al., 2019).

### Lysine

The first step of lysine catabolism is located in the cytosol where lysine is converted into 2-aminoadipate-6-semialdehyde (AASA). The next enzyme, α-aminoadipate semialdehyde dehydrogenase, can be targeted to cytosol or mitochondria (Fuchs et al., 2020) and its activity produces α-aminoadipate and NADH (Arruda and Barreto, 2020). D-2-hydroxyglutarate (D-2-HG) is most likely to be generated from lysine products inside mitochondria due to metabolic analysis of plants growing in media supplemented with different possible precursors (Engqvist et al., 2011). Mitochondrial D-2-HG dehydrogenase (D-2HGDH) oxidizes 2-HG and donates electrons to the ETC through the ETF/ETFQO complex (Araújo et al., 2010). Hence, it is possible either AASA or 2-HG enter mitochondria as direct respiratory substrates. 2-HG accumulation is observed in D-2HGDH mutants and during environmental stress in plants. This accumulation might inhibit transaminases and transporters involved in the usage of other respiratory substrates (Engqvist et al., 2011). Interestingly, D-2HGDH is in the same co-expressing network as several genes involved in β-oxidation and degradation of amino acids and chlorophyll (Engqvist et al., 2009). The generation of 2-OG from lysine via D-2HGDH is important to support TCA cycle activity as it is used to enable normal respiration and biosynthesis in NAD- and NADP-isocitrate dehydrogenase mutants (Boex-Fontvieille et al., 2014).

### Formate

Formate is known to be produced during abiotic stresses such as hypoxia, aluminum toxicity, and low pH, and its degradation is linked to mitochondrial metabolism (Nunes-Nesi et al., 2014; Lou et al., 2016). Formate found in plants which have been exposed to stress could be synthesized during photorespiration from serine, arise from methanol metabolism, or be made from glyoxylate (Alekseeva et al., 2011). Formate is mainly metabolized by mitochondrial formate dehydrogenase (FDH) and formate accumulation is known to induce the expression of FDH (Hourton-Cabassa et al., 1998; Shingaki-Wells et al., 2014). The activity of FDH generates NADH inside the mitochondrial matrix which can be used by the electron transport chain and coupled to ATP synthesis and CO_2_ release (Alekseeva et al., 2011).

## Potential respiratory substrates

There are also other plant metabolites which could be important electron donors to the mitochondrial electron transport chain. But catabolic pathways of these metabolites have not been identified in mitochondria, are disputed, or parts of them are located outside the mitochondria. Even if they are not direct substrates, we contend they could be exploited more efficiently for respiration by future bioengineering owing to their availability in cells.

### Chlorophylls

Chlorophyll breakdown is a pronounced sign of senescence, carbon and/or nitrogen starvation, and other stresses. It allows the recycling of nitrogen and possibly carbon from the chlorin ring and the prenyl side chains (Hörtensteiner and Kräutler, 2011; Gutbrod et al., 2019). After magnesium removal, the chlorin ring is converted into pheophorbide a and then to non-fluorescent chlorophyll catabolites which accumulate in the vacuole (Pruzinská et al., 2005; Hörtensteiner and Kräutler, 2011). On the other hand, mass-spectrometry experiments indicate the main breakdown products of the prenyl side chain are phytols (Mach, 2015). Although studies have suggested that the phytol degradative pathway can provide substrates for respiration during sugar starvation (Ishizaki et al., 2005), components of the phytol catabolic pathway have not been identified (Durrett and Welti, 2021). Phytol might be degraded to phytenal which accumulates in senescing oat leaves and then to phytanoyl-CoA via unknown steps (Ishizaki et al., 2005). Mutations in a gene encoding ETFQO lead to accumulation of phytanoyl-CoA which results in the inability of plants to survive extended darkness. This suggests phytanoyl-CoA might be an alternative electron donor for the ETC via ETF/ETFQO (Ishizaki et al., 2005).

In mammalian catabolic pathways, phytanoyl-CoA undergoes α-oxidation and β-oxidation in peroxisomes and mitochondria (Kohlmeier, 2015; Durrett and Welti, 2021; Gutbrod et al., 2021). In plants, phytanoyl-CoA hydroxylase and 2-hydroxy-phytanoyl-CoA lyase have been localized to peroxisomes and characterized to be the main pathway of phytanoyl-CoA metabolism (Araújo et al., 2011a; Yang et al., 2022). While plant mitochondria do not contain β-oxidation pathways, it has been suggested that isobutyryl-CoA made from peroxisomal oxidation of phytanoyl-CoA could be a substrate of IVDH in plant mitochondria (Däschner et al., 2001; Yang et al., 2022). In this case, isobutyryl-CoA may enter the mitochondria and act as a respiratory substrate by contributing electrons to the ubiquinol pool via ETF/ETFQO leading to oxygen consumption and ATP synthesis. Evidence for such transport and the identity of a mitochondrial isobutyryl-CoA transporter is still lacking (Hildebrandt et al., 2015; Hildebrandt, 2018). In addition to isobutyryl-CoA, earlier steps in phytol metabolism can result in the production of acetyl-CoA, succinate, and formate (Kohlmeier, 2015) that can each act as direct respiratory substrates via known pathways as discussed previously.

### Acetate

Acetate is well known as a respiratory substrate in many microorganisms (De Mets et al., 2019), but it is often overlooked in consideration as a respiratory substrate in plants. Acetate is readily made during carbon metabolism in plant cells, rising to concentrations of up to 1 mM in green tissues (Bodo and Stumpf, 1982) and Arabidopsis seedlings can tolerate and use exogenously supplied acetate as a carbon source (Lin and Oliver, 2008). Recently, feeding acetate to lettuce (*Lactuca sativa*) showed how acetate can be incorporated into biomass and support energy production (Hann et al., 2022). However, acetate cannot be considered as a direct respiratory substrate owing to the lack of a plant mitochondrial acetyl-CoA synthetase (ACS; Fu et al., 2020). Exogenous application of isotopically labeled acetate to plants shows labeled carbon rapidly enters the TCA cycle (Lin and Oliver, 2008) which suggests acetate is converted into acetyl-CoA in plastids and peroxisome. Acetyl-CoA then enters the mitochondria as a respiratory substrate. Knocking out both the plastid ACS and the peroxisomal short-chain acyl-CoA synthetase (acetate non-utilizing1; ACL1) (Fu et al., 2020) leads to increased endogenous acetate levels, delayed growth and sterility while the loss of only one does not cause any aberrant phenotype (Turner et al., 2005; Sofeo et al., 2019). ACL1 is essential for the incorporation of carbon from acetate to TCA intermediates while ACS incorporates carbon into leucine and fatty acids via acetyl-CoA (Pracharoenwattana et al., 2005; Fu et al., 2020). This probably explains the pathway of acetate entry to mitochondria and its use to fuel respiration. A role of acetate in plant metabolism is most prominent in conditions of hypoxic or drought stress when enzymes for fermentation such as pyruvate decarboxylase and acetaldehyde dehydrogenase are induced and mtPDC is restricted (Oliver et al., 2009; Mithran et al., 2014; Kim et al., 2017). These enzymes, together with plastid ACS, constitute a “pyruvate dehydrogenase shunt” that can decarboxylate pyruvate via acetate to acetyl-CoA, independently of PDC (Aslan et al., 2017). The operation of a PDC bypass in leaves has been proposed as an adaptive response to episodic thiamin diphosphate deficiency which leads to temporary loss of PDC activity (Joshi et al., 2019). Hence, acetate catabolism could have an indirect role as a substrate for mitochondrial respiration via acetyl-CoA-linked metabolism in other cellular compartments under both normal conditions and environmental stress.

### Serine

Serine is generally considered as a product of mitochondria rather than as a respiratory substrate owing to the lack of plant mitochondrial serine catabolic pathways. Serine is made in abundance from glycine during photorespiration in the light and a large proportion is transported via the cytosol to the peroxisome to complete the cycle, with some removal for cysteine synthesis (Bonner et al., 2005). Serine can also be made from glycolytic intermediates in the cytosol or plastid and can be imported into mitochondria independently of the photorespiratory cycle (Haas et al., 2008; Kory et al., 2018). Remarkably, serine can reach concentration >10 mM in the cytosol and in phloem sap (Igamberdiev and Kleczkowski, 2018) which is the highest concentration of any amino acid after glutamate and aspartate. A plant serine dehydratase (serine racemase) in the cytosol can generate pyruvate from serine (Fujitani et al., 2006) which can enter mitochondria and contribute to respiration. The efficiency of using serine as a mitochondrial pyruvate source would be low if serine had to be exported from mitochondria and re-enter as pyruvate due to transport logistics and the presence of competing pathways in the cytosol.

## Opportunities to generate bypasses to prioritize certain respiratory substrates

Synthetic bypasses have been both theorized and then implemented to improve photosynthesis, photoprotection, and shortcut photorespiration with the aim to increase plant yield (Hagemann and Bauwe, 2016; Eisenhut et al., 2019; Kubis and Bar-Even, 2019; De Souza et al., 2022). This has inspired theoretical re-designing of aspects of respiration under appropriate conditions to generate the same amount of ATP for less carbon loss or to lower maintenance ATP costs of the cell to decrease the need for current rates of respiration (Amthor et al., 2019). Apart from avoiding futile cycles and optimizing alternative respiration pathways (Amthor et al., 2019), a missing piece of this theory is which respiratory substrates should be prioritized for use in optimal respiration. An optimal system would use the appropriate substrates at the right time so that plant mitochondria can oxidize readily available substrates for energetic benefits rather than releasing carbon as CO_2_ from compounds that are energetically expensive to make in the first place. Energetic accounting to generate energetic profiles (Figure 5) could include weighing the reductant and ATP yield of substrates against their carbon loss and oxygen utilization stoichiometries (Figure 5, A and B from the calculations in Supplemental Table 1) and considering timing or circumstances under which they would be available in plants for use (Figure 5C). For example, we note that acetate and serine have similar energetic use profiles to the major respiratory substrates pyruvate, glycine, alanine, and 3-PGA suggesting they could be future respiratory substrates without lowering energetic efficiency per C or per O (Figure 5A). Acetate can become a direct respiratory substrate by introducing a mitochondrial acetyl-CoA synthetase and a suitable monocarboxylate transporter. The introduction of a mitochondrial serine dehydratase could stimulate the usage of serine for respiration. Similarly, introduction of an isobutyryl-CoA transporter could directly link phytol catabolism with mitochondrial respiration (Figure 4). The introduction of these pathways can be combined in a synthetic metabolon to direct the resulting pyruvate and acetyl-CoA flux into ATP production.

Evidence for MPC-PDC and MDH-CS-ACO channeling, and the apparent existence of separate metabolic pools within mitochondria, indicates that mitochondrial regulation of whether substrates are oxidized for respiration or are used to make biosynthetic reserves is much more sophisticated than it has been considered in the past. The identification of more channels and regulatory circuits will allow smarter metabolic manipulation than is possible today. This knowledge will allow artificially generated metabolic channels to be built to introduce metabolic activities to mitochondria to oxidize substrates, or simply to increase transport of certain metabolites to allow participation in wider parts of cellular catabolism (Venayak et al., 2015). Designing such systems needs the knowledge of how channels are or could be formed and regulated, and energetic accounting to determine which direct substrates should be prioritized for plant respiration. Metabolons can be formed either by physical interaction of proteins (Zhang and Fernie, 2021) or simply by co-localization to mediate the passing of intermediates from one enzyme to the next (Le et al., 2022). There are strategies to artificially create these microenvironments of sequential enzymes in the mitochondria, for example by introducing nanoparticles to bind sequential enzymes or enzyme encapsulation in virus-like particles (Pröschel et al., 2015). Channel regulation will also be important to optimize dynamic metabolic shifts at specific developmental stages or stresses, enabling the turnoff and on of metabolic channeling as required. A recent implementation of metabolic channel regulation was made to dynamically alter malate synthesis through assembling PDH and ICL to cluster the two substrates for malate synthase. CRISPR–Cas6 family proteins and RNA scaffolds were repurposed to trigger a tripling of malate synthesis rate (Mitkas et al., 2022). Understanding how plant respiration is controlled and controlling it in ways is within our grasp and will enable this foundational metabolic pathway to be an important part in future plant improvement (see Outstanding Questions).

ADVANCES

- The input substrates of respiration vary markedly between plant tissues and under different environmental conditions.
- Mitochondrial transporters for carboxylic acids have been characterized recently, expanding our understanding of how mitochondria access respiratory substrates.
- Respiratory substrates can arise from multiple sources inside cells; for example, pyruvate can be produced from phosphoenolpyruvate, alanine, and malate, and respiration via each source has a different usage preference.
- Plant cells regulate the use of respiratory substrates via post-translational processes including formation of metabolons or metabolic domains.
- Amino acids that accumulate in plants during stresses have dual use: to help meet energetic demands during recovery and to provide protection against ROS and osmotic stress.

OUTSTANDING QUESTIONS

- What are the plant mitochondrial transporters for alanine, proline, glycine, and oxo-acids resulting from BCAA metabolism?
- To maximize energetic benefits for plants (ATP output per resource input), which substrates are best for complete catabolism to CO_2_ and which are best for partial oxidation to reserve carbon for biosynthesis?
- How important are metabolons, microenvironments, and mitochondrial heterogeneity in regulating substrate choice and usage in plant respiration?
- How can we best engineer the usage of alternative respiratory substrates in plants and test the benefits for whole plant carbon use efficiency and growth?

## Supplemental data

The following materials are available in the online version of this article.

**Supplemental Table S1**. Energetic characteristics, stoichiometry of catabolic products, and concentrations of respiratory substrates.

## Supplementary Material

kiac599_Supplementary_Data
